# Genetic Variations of Vitamin A-Absorption and Storage-Related Genes, and Their Potential Contribution to Vitamin A Deficiency Risks Among Different Ethnic Groups

**DOI:** 10.3389/fnut.2022.861619

**Published:** 2022-04-28

**Authors:** Masako Suzuki, Meika Tomita

**Affiliations:** Department of Genetics, Albert Einstein College of Medicine, Bronx, NY, United States

**Keywords:** retinoid, vitamin A deficiency, genetic variations, micronutrient, ethnicity

## Abstract

Vitamin A, an essential fat-soluble micronutrient, plays a critical role in the body, by regulating vision, immune responses, and normal development, for instance. Vitamin A deficiency (VAD) is a major cause of xerophthalmia and increases the risk of death from infectious diseases. It is also emerging that prenatal exposure to VAD is associated with disease risks later in life. The overall prevalence of VAD has significantly declined over recent decades; however, the rate of VAD is still high in many low- and mid-income countries and even in high-income countries among specific ethnic/race groups. While VAD occurs when dietary intake is insufficient to meet demands, establishing a strong association between food insecurity and VAD, and vitamin A supplementation is the primary solution to treat VAD, genetic contributions have also been reported to effect serum vitamin A levels. In this review, we discuss genetic variations associated with vitamin A status and vitamin A bioactivity-associated genes, specifically those linked to uptake of the vitamin in the small intestine and its storage in the liver, as well as their potential contribution to vitamin A deficiency risks among different ethnic groups.

## Introduction

Malnutrition refers to deficiencies, excess, or imbalance of energy or nutrition intake. In 2020, about one in three people in the world (2.37 billion) did not have access to adequate food (1). Iron, iodine, folate, vitamin A, and zinc deficiencies are the most significant micronutrient-related malnutrition conditions in the world, specifically in low-income and middle-income countries (2–4). Vitamin A, an essential fat-soluble micronutrient, plays a critical role in the body, effecting vision, immune responses, and normal development. Vitamin A deficiency (VAD) is a major cause of xerophthalmia and increases the risk of death from infectious diseases. In addition, prenatal exposure to VAD is associated with preterm birth, lung and kidney functions of the fetus, and mortality of offspring born to mothers with human immunodeficiency virus infections (5–7). While VAD prevalence rates have reduced in the last two decades, about 30% of children world-wide under the age of 5 years are still vitamin A deficient, and 1.7% of all deaths are attributable to VAD in this age group (3). VAD occurs when dietary intake is insufficient to meet demands; thus, food insecurity is strongly associated with its prevalence. The amount of vitamin A we need in the body depends on age and sex. The Recommended Daily Allowances (RDAs) recommended by FDA is 900 μg of Retinol Activity Equivalents (RAE) for adult men, 700 μg for adult women, 770 μg for pregnant women, 1300 μg for breastfeeding women, and 400 μg for infants aged 6 months or less (8). An RDA is the average daily dietary intake level to meet sufficient nutrition requirements for nearly all (97–98%) healthy individuals in a group. RAE for each vitamin A form can convert as: 1 μg RAE = 1 μg retinol, 2 μg β-carotene from a supplement, 12 μg β-carotene from food, 24 μg α-carotene from food, or 24 μg β-cryptoxanthin from food (8, 9). There are two major forms of vitamin A in the human diet, preformed vitamin A, mainly as retinol, retinyl esters and small amount of retinoic acid, and provitamin A carotenoids, mainly as β-carotene. The proportion of preformed vitamin A and provitamin A carotenoid intake is dependent on our diet. Preformed vitamin A is derived from animal products and provitamin A carotenoids from plant-derived food [reviewed in (10)]. Since our body can downregulate bioconversion of provitamin A carotenoids (11, 12), high intakes of fruit and vegetables will usually not significantly contribute to hypervitaminosis A. However, it has been reported that high-dose supplementation of β-carotene increases cancer incidence in the lung and stomach (13–16), suggesting that excess supplementation of provitamin A carotenoid could be harmful to humans. In contrast to provitamin A, if the dietary intake of preformed vitamin A becomes high through foods, such as animal liver, fortified foods, or supplements, the body will store the excess in the liver, reaching levels defining hypervitaminosis A (defined as ≥1 μg/g liver) (17, 18). Ingested preformed vitamin A and provitamin A carotenoid are processed for normal physiological functions or storage (bioavailability). While the metabolic pathways by which each form of vitamin A is metabolized are partly different until they are converted to retinal, both preformed vitamin A and provitamin A carotenoids are metabolized to the active vitamin A molecules, retinoic acid that directly regulates gene activity and function, in the body [reviewed in (19)].

Although vitamin A deficiency in high-income countries is believed to be a rare condition, it has been reported that certain ethnic groups have high rates of vitamin A deficiency even in developed countries (20–22). Several genetic variations to play a role in serum vitamin A levels have been reported in humans (23, 24). As we reported previously, the allele frequencies of some genetic variations associated with vitamin A bioavailability vary among ethnic groups (22). Moreover, Obrochta et al. reported that strain and tissue-specific variations of retinol and all trans-retinoic acid in serum and tissues of five inbred mouse strains feeding two different vitamin A concentration diets to the mothers (25). Since all mouse strains fed equal amounts of vitamin A in the study, their findings suggest the existence of genetic variation-dependent vitamin A variations. This review focuses on genetic variations associated with vitamin A status and vitamin A bioactivity-associated genes, specifically vitamin A uptake in the small intestine and storage in the liver, and their potential contribution to vitamin A deficiency risks among different ethnic groups.

## Intestinal Absorption of β-Carotene

In the human diet, retinyl palmitate and β-carotene are the dominant forms of vitamin A (26). While retinyl palmitate is enzymatically converted to retinol in the intestinal lumen before absorption by enterocytes, β-carotene is partially converted to retinol in the enterocytes. Interestingly, high inter-individual variability in carotenoid absorption from nutritional intake has been observed in human studies (27–30). Moreover, reported β-carotene absorption rates differ between individuals as well as between studies, for instance, 3.4% (*n* = 12 individuals) (30) to 90.0% (*n* = 5 individuals) (28) following the oral administration of a pharmacologic dose of β-carotene. These interindividual efficiency ranges were much higher than that of the preformed vitamin A (retinol) absorption efficiency (70 to 90%) (8, 31). Intriguingly, O'Neill and Thurnham further reported that while the interindividual variations of absorption rates of β-carotene are high, the response to β-carotene was constant within an individual and reproducible over time (30). De Pee & West reviewed and noted that a number of factors influence the bioavailability of carotenoids, which they grouped in the mnemonic SLAMENGHI: Species of carotenoids, molecular Linkage, Amount of carotenoids consumed in a meal, Matrix in which the carotenoid is incorporated, Effectors of absorption and bioconversion, the Nutrient status of the host, Genetic factors, Host-related factors, and mathematical Interactions (32). The genetic contribution to interindividual variability of β-carotene status has also been proposed [reviewed in (33)]. While a part of β-carotene incorporated in mixed micelles reaches the apical membrane as free molecules, the scavenger receptor class B type I (SR-BI) protein, encoded by *SCARB1*, and cluster determinant 36 (CD36), also known as FAT (fatty acid transporter), are involved in cellular uptake of β-carotene in the small intestine (29, 34). Associations between SNPs and haplotypes in *SCARB1* and *CD36* and plasma concentrations of provitamin A carotenoids have been identified (29, 35). Study participants bearing the C allele of the SNP rs61932577 at the *SCARB1* intron 5 had lower β-carotene concentrations than men homozygous for the T allele, while participants bearing rs5888C/rs4238001C/rs61932577T haplotype had lower plasma β-cryptoxanthin than the participants did bearing the rs5888T/rs4238001C/rs61932577C haplotype (35). The same research group also reported that the *CD36* haplotype affects plasma provitamin A carotenoid (29).

**Figure 1:**
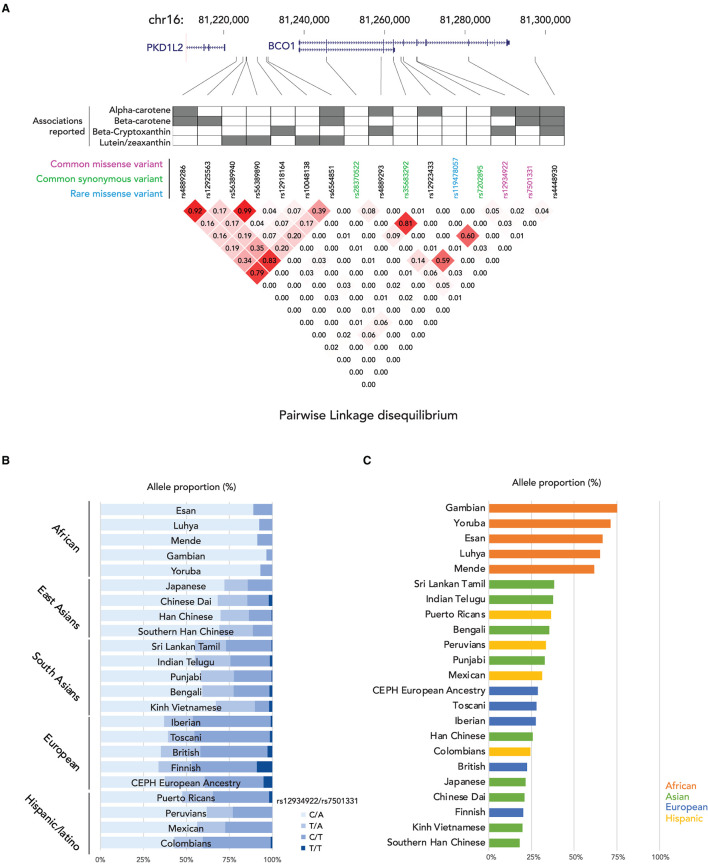
Summary of genetic variations of *BCO1* gene. **(A)** (top) locations of genetic variants in BCO1 locus; (middle) the association to the provitamin A status of each genetic variation; (bottom) Linkage Disequilibrium (LD) plot, the numbers in the boxes indicate the LD status and darker red shading illustrate higher LD; **(B)** Proportions of *BCO1* gene activity genotypes by ethnic groups. **(C)** Proportion of low *BCO1* gene activity haplotype defined by Hendrickson's estimation by ethnic groups.

In the enterocyte, the β-carotene taken up is converted into retinaldehyde by the β-Carotene 15,15′ oxygenase 1 enzyme [BCO1, previously named β-Carotene 15,15'-monooxygenase 1 (BCMO1)] and the retinaldehyde is then reduced to retinol by a retinal reductase (36, 37). We summarize the known genetic polymorphisms of the *BCO1* gene, the associations of retinoid status, and the linkage disequilibrium of the known genetic polymorphisms in Figure 1A. It has been suggested that the reduced enzymatic activity of BCO1 might cause the large interindividual variability of β-carotene absorption as a consequence of genetic polymorphisms in the gene (38). Importantly, the study reported that these polymorphisms, rs12934922 A/T (R267S) and rs7501331 C/T (A379V), are common variants (i.e., variants with a frequency of >1% in the population). They reported that 379V and 267S + 379V reduced the ability to convert β-carotene (38). Using the 1,000 Genomes Project dataset, we found that the low BCO1 activity genotype allele frequency is higher in European Ancestry, especially the Finnish group who have the highest percent (267S + 379V 9.09, and 379V 37.9%) out of all 25 ethnic/racial groups (Figure 1B). The aforementioned study which found that β-carotene supplementation increases lung cancer incidence was conducted on Finnish cohorts suggesting a subset of participants carry low BCO1 activity genotype (15, 16). A *Bco1* knock-out mouse model study showed that while β-carotene supplementation to mice lacking Bco1 function reduced the inflammatory response, it increased concentration of β-carotene in lung and serum and retinyl ester concentration in the lung compared to wild type mice (39). This result suggests that a person who carries the genotype that reduces the activity of the Bco1 enzyme may more readily accumulate β-carotene and retinyl ester in the lung following β-carotene supplementation. Therefore, assessing *BCO1* genotypes in participants who developed lung cancer in the β-carotene supplementation study is needed to test whether the *BCO1* genotype also modulates lung cancer incidence. The associations between other common variants of the gene and genes adjacent to the *BCO1* gene and serum β-carotene status also have been extensively reported (40–45). The rs6564851 common variant located about 8 kb upstream of the *BCO1* gene is reported to be associated with circulating levels of β-carotene in different ethnic groups (40, 43, 44). The rs6564851 variant is located within a haplotype that includes other genetic polymorphisms (Figure 1A). Hendrickson et al. carefully tested the associations between *BCO1* common variants and plasma carotenoid concentrations and developed gene score systems that predict plasma carotenoid concentrations in women of European descent (42). In their model, the rs12934922 T allele and rs4889286 T allele predict higher plasma β-carotene (42). Interestingly, the proportions of these allele combinations vary between ethnic/race groups, with the allele frequencies of the “lower” plasma β-carotene combination highest among African ancestry groups (Figure 1C). These findings show that testing the associations of genotypes with plasma β-carotene in a multi-ethnic population is needed. Besides the common variants, one missense pathogenic variant of *BCO1*, rs119478057, has been identified in a patient with hypercarotenemia and hypovitaminosis A (46, 47). The rs119478057 T allele is a rare variant with a frequency less than 0.01 in the European population.

β-carotene-9',10'-oxygenase 2 (BCO2) is another carotenoid converting enzyme located in the mitochondria; it cleaves β-carotene asymmetrically in position 9',10' generating long-chain apocarotenoids. BCO1 and BCO2 also differ for their substrate specificity. Lutein, lycopene, β-cryptoxanthin, and zeaxanthin are all specific substrates for BCO2, which BCO1 cannot cleave (48). While livestock studies suggested that BCO2 mutations are associated with carotenoid metabolism alterations, genetic variation associations in humans' carotenoid statuses are not elucidated yet. Table 1 lists the reported genetic variants and the reported associations on provitamin A intestinal absorption.

**Table 1:** A list of sequence polymorphisms and the reported associations on provitamin A intestinal absorption.

**Gene symbol**	**dbSNP ID**	Position^*^	**Allele**	**Reference allele frequency (1000 genome)**	**Type**	**Reported associations on serum provitamin A concentrations**	**Reference**
SCARB1	rs61932577 rs5888 rs4238001	chr12:124811816 chr12:124800202chr12:124863717	G/A,C,T A/G,T C/T	0.964856 0.322883 0.935703	Intron variant Synonymous variant Missense variant	Plasma provitamin A carotenoids (high in rs61932577 TT), beta-cryptoxanthin [high in TCC haplotype (rs5888, rs4238001, rs61932577)]	Borel et al. (29)
CD36	rs1984112	chr7:80613604	A/G	0.653155	Intron variant	Beta-cryptoxanthin (high in women AA), alpha-carotene [high in GGACC haplotype (rs1984112, rs1761667, rs1527479, rs1527483, rs13230419)], beta-cryptoxanthin [high in GGACC haplotype (rs1984112, rs1761667, rs1527479, rs1527483, rs13230419)]	Borel et al. (29)
	rs1761667	chr7:80615623	G/A	0.609625	Intron variant	Beta-cryptoxanthin (high in women AA), alpha-carotene [high in GGACC haplotype (rs1984112, rs1761667, rs1527479, rs1527483, rs13230419)], beta-cryptoxanthin [high in GGACC haplotype (rs1984112, rs1761667, rs1527479, rs1527483, rs13230419)]	
	rs1527479	chr7:80643252	T/A,C	0.650958	Intron variant	Alpha-carotene [high in GGACC haplotype (rs1984112, rs1761667, rs1527479, rs1527483, rs13230419)], beta-cryptoxanthin [high in GGACC haplotype (rs1984112, rs1761667, rs1527479, rs1527483, rs13230419)]	
	rs1527483	chr7:80672184	G/A	0.898163	Intron variant	Alpha-carotene [high in GGACC haplotype (rs1984112, rs1761667, rs1527479, rs1527483, rs13230419)], beta-cryptoxanthin [high in GGACC haplotype (rs1984112, rs1761667, rs1527479, rs1527483, rs13230419)]	
	rs13230419	chr7:80679969	C/T	0.65615		Alpha-carotene [high in GGACC haplotype (rs1984112, rs1761667, rs1527479, rs1527483, rs13230419)], beta-cryptoxanthin [high in GGACC haplotype (rs1984112, rs1761667, rs1527479, rs1527483, rs13230419)]	
	rs7755	chr7:80676955	G/A	0.612021	3' UTR variant	Beta-cryptoxanthin (high in GG women and AA men)	
BCO1	rs4889286	chr16:81223108	C/T	0.507987		Alpha-carotene (high in T allele), beta-carotene (high in T allele)	Hendrickson et al. (42)
	rs12925563	chr16:81224725	T/C,G	0.488618		Beta-carotene^†^	
	rs56389940	chr16:81225547	C/A,T	0.846046		Lutein/zeaxanthin (high in A allele)	
	rs56389890	chr16:81225661	G/T	0.844649		Lutein/zeaxanthin^†^	
	rs12918164	chr16:81228347	G/A	0.826478		Beta-Cryptoxanthin (high in A allele)	
	rs10048138	chr16:81230572	A/C,G,T	0.278355		Lutein/zeaxanthin (high in A allele)	
	rs6564851	chr16:81230992	T/G	0.476238		Alpha-carotene (high in G allele), beta-carotene (high in G allele), beta-Cryptoxanthin (high in G allele), lutein/zeaxanthin (high in T allele)	Hendrickson et al., He et al., Ferrucci et al. (40, 42, 45)
	rs4889293	chr16:81259125	C/A, G	0.761581		Alpha-carotene (high in G allele), beta-Cryptoxanthin (high in G allele)	Hendrickson et al. (42)
	rs12923433	chr16:81264097	A/G	0.747804	Intron variant	Alpha-carotene^†^	Hendrickson, et al. (42)
	rs119478057	chr16:81264677	C/A,T	0.999601	Missense variant	Hypovitaminosis A (T170M mutation)	Lindqvist, et al. (46)
	rs12934922	chr16:81268089	A/G, T	0.772764	Missense variant	Alpha-carotene (high in T allele), beta-carotene (high in T allele), beta-Cryptoxanthin (high in T allele), lutein/zeaxanthin (high in T allele), retinol^†^	Hendrickson et al., Leung et al. (38, 42)
	rs7501331	chr16:81280891	C/T	0.847843	Missense variant	Alpha-carotene (high in C allele), beta-carotene (high in C allele)	Leung et al., He et al., Hendrickson et al. (38, 42, 45)
	rs4448930	chr16:81297397	G/A,C,T	0.822484		Beta-carotene (high in C allele), beta-Cryptoxanthin (high in C allele)	Hendrickson et al. (24)

* *hg38*;

† *allelic information was not available*.

## Intestinal Absorption of Retinol

Dietary retinyl esters are enzymatically converted to retinol in the intestinal lumen before absorption by enterocytes. Pancreatic lipase, encoded by *PNLIP*, and pancreatic lipase-related protein 2, encoded by *PNLIPRP2*, hydrolyze retinyl palmitate to retinol (49). *PNLIP*, on chromosome 10q26.1, encodes a 465-amino acid protein and is predominantly expressed in the pancreas (50). One missense variant of *PNLIP* (rs746000327 C/T) associated with pancreatic lipase deficiency has been identified in two brothers from a consanguineous family of Arab ancestry (51). The frequency of rs746000327 T allele is less than 0.0001 in NHLBI Trans-Omics for Precision Medicine (TOPMed, PRJNA400167) Whole Genome Sequencing (WGS) Project and the Genome Aggregation Database (gnomAD, PRJNA398795). Currently, no sequence variations with clinical significance on *PNLIPRP2* have been reported. In enterocytes, retinol that has been taken up binds to retinol-binding protein 2 [RBP2; initially called cellular retinol-binding protein, type II (CRBPII) (52)]. This retinol-RBP2 complex serves as a substrate for microsomal lecithin:retinol acyltransferase (LRAT). *RBP2* is exclusively expressed in the small intestinal absorptive cells with specific localization (highly expressed in duodenum and jejunum and less expressed in the ileum). It is one of the most abundant soluble proteins in the small intestine (53, 54). It has been suggested that the expression of *RBP2* is regulated predominantly by dietary fatty acids but little by dietary retinoids, as reviewed by Takase et al. (55). Currently, no sequence variations with clinical significance have been identified in *RBP2*. Retinol bound to RBP2 converted from retinyl palmitate is re-esterified by the enzyme lecithin:retinol acyltransferase (*LRAT*) and acyl coA:retinol acyltransferase (*ARAT*). While retinol complexed with CRBP2 is the preferred substrate of LRAT, uncomplexed retinol may be esterified by ARAT. LRAT may mainly esterify in the intestine when the retinol is present in normal amounts. In contrast, when retinol is present at high levels, and CRBP becomes saturated, ARAT may function to esterify the excess (26). Three rare pathogenic variants of *LRAT* have been identified in European populations; a single nucleotide mutation, rs104893848, and two 2-bp deletions, rs761717462 and rs1560870755. Two variants, rs104893848 and rs761717462, were identified as early-onset severe retinal dystrophy-associated mutations (56). The rs104893848 is a missense variant causing the Ser175Arg (S175R) amino acid change. The rs761717462 variant is a 2-bp deletion (396delAA) which causes a frameshift after codon 133 to encode 11 amino acids unrelated to the wildtype sequence followed by a premature stop codon. The S175R missense variant shows no acyltransferase activity in an *in vitro* study (56). The third variant, rs1560870755, a Leber congenital amaurosis-associated mutation, is a mutation causing a frameshift at codon 73 and a premature termination codon at residue 120. The mutation results in the loss of essential amino acids for accepting the acyl group from the lecithin (57). In humans, two transferases can also have acyl-CoA:retinol acyltransferase activities have been identified, diacylglycerol O-acyltransferase 1 (*DGAT1*) (58–60) and acyl-CoA wax alcohol acyltransferase 2 (*AWAT2*) (61). While both genes have the acyl-CoA:retinol acyltransferase activities, DGAT1 plays an important intestinal ARAT *in vivo* (60). One pathogenic variant of *DGAT1*, rs148665132, has been identified in a study of a family of Ashkenazi Jewish descent with congenital diarrheal disorders; however, the effect of the variant on acyl-CoA:retinol acyltransferase activity is not yet elucidated (62). Table 2 lists these pathogenic sequence polymorphisms of intestinal retinol absorption-related genes.

**Table 2:** A list of pathogenic sequence polymorphisms of intestinal retinol absorption related genes.

**Gene symbol**	**dbSNP ID**	Position*	**Allele**	**Reference allele frequency (1000 genome)**	**Type**	**Reported diseases**	**Reference**
PNLIP	rs746000327	chr10:116555268	C/T	0.999996	Missense variant	Pancreatic lipase deficiency	Behar et al. (51)
LRAT	rs104893848	chr4:154744851	T/A,C	0.999944	Missense variant	Early-onset severe retinal dystrophy	
	rs761717462	chr4:154744723-154744727	delAA	0.999996	Frameshift variant	Early-onset severe retinal dystrophy	Thompson et al. (56)
	rs1560870755	chr4:154744543-154744544	delAT	-	Frameshift variant	Leber congenital amaurosis	

## Transport, Absorption, and Storage In The Liver

As mentioned earlier, newly absorbed vitamin A in the small intestine is packaged into chylomicrons and secreted to the lymphatic system by enterocytes. Once the chylomicrons reach the circulation, a part of the triglycerides are hydrolyzed, and the lipoprotein particles shrink to form chylomicron remnants. About 70% of retinoids packaged into chylomicron remnants in the bloodstream are taken up by the liver, the main organ involved in storing retinoids and regulating serum vitamin A levels (63, 64). Besides the liver, chylomicron remnants are delivered to the lung, heart, kidney, adipose tissue, muscle, spleen, and bone marrow (65, 66). In the liver, hepatocytes play the leading role in the uptake of chylomicrons, where retinyl ester is hydrolyzed again to form retinol by several enzymes, such as carboxyl ester lipases and carboxylesterases, and hepatic lipases [reviewed in (67)]. In the hepatocyte, formed retinol is bound to cellular retinol-binding protein 1 (CRBP1 or RBP1) and then is transferred to retinol-binding protein 4 (RBP4 or RBP) to secrete into e the circulation or transferred to hepatic stellate cells where retinol is re-esterified by LRAT. From the hepatocyte, retinol bound to the RBP4 is secreted into the circulation and delivered to the target cells and organs. RBP4 is the sole specific retinol-binding protein in the blood, and RBP-bound retinol accounts for approximately 99% of all retinoids present in the blood in the fasting state (68). Genetic variations of *RBP4* and serum retinol levels have been identified in European populations and estimated that 2.3% of the variance in serum retinol levels in the cohort are accounted for the genetic variations of *RBP4* and transthyretin (*TTR*)(24), and low serum retinol level allele frequency varies between race/ethnic groups (22). Delivery of retinol to the target cells is mediated by the binding of retinol-bound- (holo-) RBP and an RBP receptor called STRA6 (Stimulated by Retinoic Acid 6) (69). STRA6 is ubiquitously expressed, and its expression status is regulated by the retinoid metabolisms (69). While there are five genetic variants listed on the NHGRI-EBI Catalog of Published Genome-Wide Association Studies, rs351242 (70), rs351237 (71), rs11635868 (72), rs10910 (73), and rs11638831 (74), no associations between these genetic variants and STRA6 function have been described. Interestingly, a missense double-nucleotide polymorphism (g.1157G>A and g.1156G>A; p.Gly304Lys, rs151341424) in *STRA6* was found in the Irish Traveler family with isolated microphthalmia and coloboma (MCOPCB8) (75), a rare autosomal recessive disorder (76). Casey reported that the G304K mutant *STRA6* protein is mislocalized and has severely reduced vitamin A uptake activity (75). Besides rs151341424, four common missense variants, rs736118, rs351236, rs971756, and rs76336272, have been identified. Of those, rs971756 and rs736118 are reported to be linked to human diseases (77–84), but associations of the variants to STRA6 function were not described.

Hepatic stellate cells play a major role in storing vitamin A in the liver. It has been estimated that approximately 70% of vitamin A in the body of healthy and well-nourished individuals is stored in the liver, specifically a small subset of cells, hepatic stellate cells (85). Hepatic LRAT expression is regulated by vitamin A status, a positive feedback loop to increase retinyl ester synthesis when cellular retinoic acid concentration is high (86–88). Two lipases mainly perform the mobilization of retinyl ester stores: the patatin-like phospholipase domain-containing 2 (PNPLA2)/adipose triglyceride lipase (ATGL) and patatin-like phospholipase domain-containing 3 (PNPLA3) proteins (89, 90). Currently, 11 genetic variants in *ATGL/PNPLA2* are reported to associate with neutral lipid storage disease with myopathy (91–94); but no associations of retinyl ester mobilization and these genetic variations have been reported. A missense genetic variation of the *PNPLA3* (I148M, rs738409) gene, one of the most prominent genetic risk factors associated with non-alcoholic liver disease (NAFLD), elevates retinyl ester levels in the liver with reduced serum retinol levels (90, 95, 96). While 32 missense variations have been identified in the *PNPLA3* gene, rs738409 is the only pathogenic genetic variant of the *PNPLA3* gene. Table 3 lists the reported genetic variants associated with the transport, absorption, and storage of vitamin A in the liver.

**Table 3:** A list of sequence polymorphisms and the reported associations on transport, absorption, and storage of vitamin A in liver.

**Gene symbol**	**dbSNP ID**	Position*	**Allele**	**Reference allele frequency (1000 genome)**	**Type**	**Reported associations on vitamin A status/metabolism**	**Reference**
RBP4	rs10882272	chr10:93588425	T/C	0.610224	Downstream variant	Homozygous for the common allele for both SNPs have higher circulating retinol levels	Mondul et al. (24)
TTR	rs1667255	chr18:31607316	A/C,G,T	0.499601			
STRA6	rs151341424	chr15:74190856-74190857	CC/TT	-	Missense Variant	Reduced vitamin A uptake activity	Casey et al. (75)
PNPLA3	rs738409	chr22:43928847	C/G	0.737819	Missense Variant	Elevates retinyl ester levels in the liver with reduced serum retinol levels	Kovarova et al., Mondul, et al., Pirazzi et al. (24, 90, 95)

## Genetic Variants and Expression Status of The Vitamin a Intake-Related Genes

**Figure 2:**
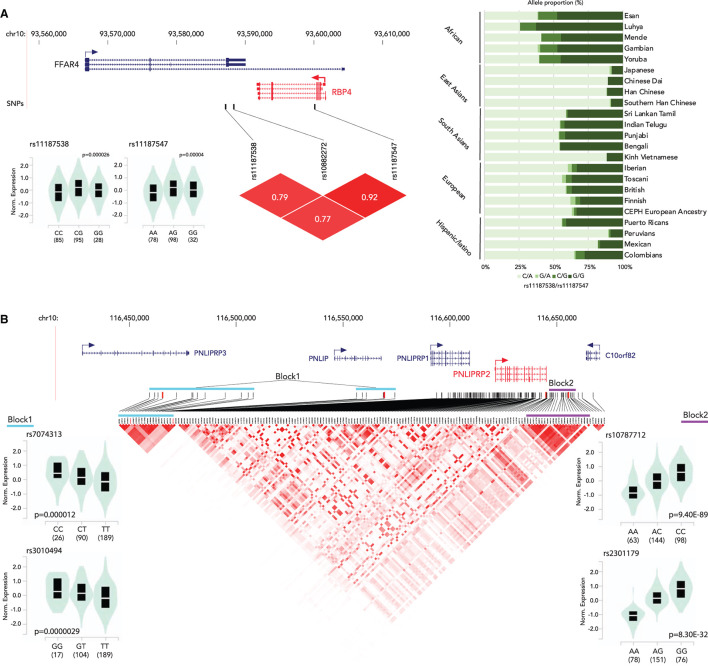
Summary of eQTLs of RBP4 **(A)** and PNLIPRP2 **(B)**. **(A)** (top) Locations of eQTLs in RBP4; (bottom left) normalized expression status of RBP4 by genotype; (bottom right) an LD plot; (right) haplotype proportion by race/ethnicity group. **(B)** (top) Locations of eQTLs in PNLIPRP2; (bottom left) normalized expression status of PNLIPRP2 by genotype (block 1); (bottom center) a Linkage disequilibrium (LD) plot of eQTLs; (bottom right) normalized expression status of PNLIPRP2 by genotype (block 2).

The associations between genetic variants and gene expression status have been well-characterized. In the previous sections, we discussed the associations of genetic variants on the vitamin A metabolism related to gene functions and human disease. This section focuses on the genetic variants that alter gene expression status. Genetic variants associated with gene expression status, expression quantitative trait loci (eQTLs), have been identified in the human genome (97–100). We searched for eQTLs of the vitamin A metabolism-related genes in the Genotype-Tissue Expression (GTEx) project database (97). We identified eQTLs clusters for *RBP4* (liver) and *PNLIPRP2* (pancreas) (Figure 2, Supplementary Table 1). The *RBP4* gene has two eQTLs, rs11187538 and rs11187547, located in a haplotype block including the previously reported genetic variant associated with lower RBP4 levels, rs10882272 (24). Allele frequency analysis showed that Eastern Asian ancestry has a higher frequency of the low-expression haplotype (rs11187538C/rs11187547A) (Figure 2A). While no genetic variants with clinical significance on *PNLIPRP2* have been reported, there are 173 eQTLs in its most highly expressed tissue, the pancreas. Haplotype analysis indicates that the *PNLIPRP2* eQTL cluster contains two large blocks (Figure 2B). The block located upstream of the gene (Block 1) is positively associated with the expression with the reference allele, and the block downstream of the gene (Block 2) is negatively associated with the reference allele. Based on the 1,000 Genomes allele frequency database, the haplotype allele frequencies of the blocks vary by race/ethnicity. Europeans and South Asians have higher frequencies with low expression haplotypes in Block 1, and East Asians have higher frequencies with low expression haplotypes in Block 2. These findings suggest that the genetic variations might contribute to the vitamin A metabolism through modifications of the expression of these vitamin A intake-related genes.

## Discussion

While the prevalence of VAD has significantly declined over recent decades (3), it remains a concern from a population health perspective that the rate of VAD remains high in many low- and mid-income countries (3, 101, 102) and in specific ethnic/race groups within high-income countries (22). West reported that approximately 127 million pre-school-aged children and 7 million pregnant women were VAD in 2003 (103). The WHO estimated in 2009 that 5.2 million preschool children and 9.8 million pregnant women were affected with the VAD-associated phenotype of night blindness (104). Many interventions have been implemented to reduce VAD in lower-income countries (101); but these interventions did not consider the individual susceptibilities associated with genetic variation and race/ethnicities. As described in this review, genetic variation near retinoid metabolism-related genes significantly contributes to the function and expression levels of these genes. Excess amounts of vitamin A can be toxic to human health and have adverse effects on normal development (reviewed in [105]). Moreover, the genome-wide association study that identified genetic variants contributing to serum retinol levels was performed in European ancestry, in which the overall rate of VAD is low (24). This raises the possibility that this kind of study may not fully detect all of the genetic variants that contribute to VAD. Although vitamin A supplementation is a primary treatment for VAD and a solution to reduce the prevalence rate of VAD in populations bearing a high prevalence of VAD, knowing the person's vitamin A metabolism-related gene variants helps to perform the treatment and intervention effectively and safely. In addition, vitamin A metabolism is not fully elucidated yet. Genetic variations associated with the process that has not been described fully could contribute to the VAD. We propose a major need to perform genome-wide association studies in multiethnic and admixed populations to fill this knowledge gap.

## Author Contributions

MT: data curation, visualization, and writing—review and editing. MS: conceptualization, visualization, writing—original draft preparation, writing—review and editing, and funding acquisition. All authors have read and agreed to the published version of the manuscript.

## Funding

This work was supported by the National Institutes of Health under award number R01HL145302. The content is solely the responsibility of the authors and does not necessarily represent the official views of the National Institutes of Health.

## Conflict of Interest

The authors declare that the research was conducted in the absence of any commercial or financial relationships that could be construed as a potential conflict of interest.

## Publisher's Note

All claims expressed in this article are solely those of the authors and do not necessarily represent those of their affiliated organizations, or those of the publisher, the editors and the reviewers. Any product that may be evaluated in this article, or claim that may be made by its manufacturer, is not guaranteed or endorsed by the publisher.

## References

[1] Food Food and Agriculture Organization of the United Nations International Fund for Agricultural Development United Nations International Children's Emergency Fund Programme WF Organization WH. The state of food security and nutrition in the world 2021. Food Agri Org. (2021), p. 240. 10.4060/cb4474en

[2] Freeland-Graves JH Sachdev PK Binderberger AZ Sosanya ME. Global diversity of dietary intakes and standards for zinc, iron, and copper. J Trace Elem Med Biol. (2020) 61:126515. 10.1016/j.jtemb.2020.126515 32450495

[3] Stevens GA Bennett JE Hennocq Q Lu Y De-Regil LM Rogers L. Trends and mortality effects of vitamin A deficiency in children in 138 low-income and middle-income countries between 1991 and 2013: a pooled analysis of population-based surveys. Lancet Glob Health. (2015) 3:e5283:15 10.1016/S2214-109X(15)00039-X 26275329

[4] Black RE Victora CG Walker SP Bhutta ZA Christian P de Onis M. Maternal and child undernutrition and overweight in low-income and middle-income countries. Lancet. (2013) 382:427–51. 10.1016/S0140-6736(13)60937-X 23746772

[5] Semba RD Miotti PG Chiphangwi JD Liomba G Yang LP Saah AJ. Infant mortality and maternal vitamin A deficiency during human immunodeficiency virus infection. Clin Infect Dis. (1995) 21:966Dis D 10.1093/clinids/21.4.966 8645848

[6] Verma RP McCulloch KM Worrell L Vidyasagar D. Vitamin A deficiency and severe bronchopulmonary dysplasia in very low birthweight infants. Am J Perinatol. (1996) 13:966–72. 8960606 10.1055/s-2007-994376

[7] Radhika MS Bhaskaram P Balakrishna N Ramalakshmi BA Devi S Kumar BS. Effects of vitamin A deficiency during pregnancy on maternal and child health. BJOG. (2002) 109:689–93. 10.1111/j.1471-0528.2002.01010.x 12118649

[8] Institute of Medicine (US) Panel on Micronutrients. Dietary Reference Intakes for Vitamin A, Vitamin K, Arsenic, Boron, Chromium, Copper, Iodine, Iron, Manganese, Molybdenum, Nickel, Silicon, Vanadium, and Zinc. Washington, DC: National Academies Press (US) (2001). 10.17226/10026 25057538

[9] West CE Eilander A van Lieshout M. Consequences of revised estimates of carotenoid bioefficacy for dietary control of vitamin A deficiency in developing countries. J Nutr. (2002) 132:2920S−6S. 10.1093/jn/132.9.2920S 12221270

[10] Harrison EH. Mechanisms involved in the intestinal absorption of dietary vitamin A and provitamin A carotenoids. Biochim Biophys Acta. (2012) 1821:70–7. 10.1016/j.bbalip.2011.06.002 21718801 PMC3525326

[11] Lobo GP Hessel S Eichinger A Noy N Moise AR Wyss A. is a retinoic acid-sensitive gatekeeper that controls intestinal beta, beta-carotene absorption and vitamin A production. FASEB J. (2010) 24:1656–656:10.1096/fj.09-150995 20061533 PMC2874479

[12] Novotny JA Harrison DJ Pawlosky R Flanagan VP Harrison EH Kurilich AC. Beta-carotene conversion to vitamin A decreases as the dietary dose increases in humans. J Nutr. (2010) 140:915–8. 10.3945/jn.109.116947 20237064 PMC2855261

[13] Tanvetyanon T Bepler G. Beta-carotene in multivitamins and the possible risk of lung cancer among smokers vs. former smokers: a meta-analysis and evaluation of national brands. Cancer. (2008) 113:150–7. 10.1002/cncr.23527 18429004

[14] Middha P Weinstein SJ MP Wein S Albanes D Mondul AM. βAM. l AM. supplementation and lung cancer incidence in the alpha-tocopherol, beta-carotene cancer prevention study: the role of tar and nicotine. Nicotine Tob Res. (2019) 21:1045–045:10.1093/ntr/nty115 29889248 PMC6636175

[15] Alpha-Tocopherol Beta Carotene Cancer Prevention Study Group. The effect of vitamin E and beta carotene on the incidence of lung cancer and other cancers in male smokers. N Engl J Med. (1994) 330:1029–35. 10.1056/NEJM199404143301501 8127329

[16] Albanes D Heinonen OP Taylor PR Virtamo J Edwards BK Rautalahti M. Alpha-Tocopherol and beta-carotene supplements and lung cancer incidence in the alpha-tocopherol, beta-carotene cancer prevention study: effects of base-line characteristics and study compliance. J Natl Cancer Inst. (1996) 88:1560–560:10.1093/jnci/88.21.1560 8901854

[17] Tanumihardjo SA Kaliwile C Boy E Dhansay MA van Stuijvenberg ME. Overlapping vitamin A interventions in the United States, Guatemala, Zambia, and South Africa: case studies. Ann N Y Acad Sci. (2019) 1446:102–16. 10.1111/nyas.13965 30265402 PMC7999526

[18] van Stuijvenberg ME Dhansay MA Nel J Suri D Grahn M Davis CR. South African preschool children habitually consuming sheep liver and exposed to vitamin A supplementation and fortification have hypervitaminotic A liver stores: a cohort study. Am J Clin Nutr. (2019) 110:91–101. 10.1093/ajcn/nqy382 31089689

[19] D2Ambrosio DN Clugston RD Blaner WS. Vitamin A metabolism: an update. Nutrients. (2011) 3:63–103. 10.3390/nu3010063 21350678 PMC3042718

[20] Hanson C Lyden E Abresch C Anderson-Berry A. Serum retinol concentrations, race, and socioeconomic status in of women of childbearing age in the united states. Nutrients. (2016) 8:508. 10.3390/nu8080508 27548213 PMC4997421

[21] Spannaus-Martin DJ Cook LR Tanumihardjo SA Duitsman PK Olson JA. Vitamin A and vitamin E statuses of preschool children of socioeconomically disadvantaged families living in the midwestern United States. Eur J Clin Nutr. (1997) 51:864–9. 10.1038/sj.ejcn.1600503 9426362

[22] Suzuki M Wang T Garretto D Isasi CR Cardoso WV Greally JM Quadro L. Disproportionate Vitamin A deficiency in women of specific ethnicities linked to differences in allele frequencies of Vitamin A-related polymorphisms. Nutrients. (2021) 13:1743. 10.3390/nu13061743 34063790 PMC8223783

[23] Gueguen S Leroy P Gueguen R Siest G Visvikis S Herbeth B. Genetic and environmental contributions to serum retinol and alpha-tocopherol concentrations: the Stanislas Family Study. Am J Clin Nutr. (2005) 81:1034–034:10.1093/ajcn/81.5.1034 15883426

[24] Mondul AM Yu K Wheeler W Zhang H Weinstein SJ Major JM Cornelis MC. Genome-wide association study of circulating retinol levels. Hum Mol Genet. (2011) 20:4724–724:10.1093/hmg/ddr387 21878437 PMC3209826

[25] Obrochta KM Kane MA Napoli JL. Effects of diet and strain on mouse serum and tissue retinoid concentrations. PLoS ONE. (2014) 9:e99435. 10.1371/journal.pone.0099435 24911926 PMC4049816

[26] Blomhoff R Green M Berg T Norum K. Transport and storage of vitamin A. Science. (1990) 250:399–404. 10.1126/science.2218545 2218545

[27] Novotny JA Dueker SR Zech LA Clifford AJ. Compartmental analysis of the dynamics of beta-carotene metabolism in an adult volunteer. J Lipid Res. (1995) 36:1825–38. 7595103

[28] Faulks RM Hart DJ Wilson PD Scott KJ Southon S. Absorption of all-trans and 9-cis beta-carotene in human ileostomy volunteers. Clin Sci. (1997) 93:585–91. 10.1042/cs0930585 9497797

[29] Borel P Lietz G Goncalves A Szabo de Edelenyi F Lecompte S Curtis P. CD36 and SR-BI are involved in cellular uptake of provitamin A carotenoids by Caco-2 and HEK cells, and some of their genetic variants are associated with plasma concentrations of these micronutrients in humans. J Nutr. (2013) 143:448–56. 10.3945/jn.112.172734 23427331

[30] O'Neill ME Thurnham DI. Intestinal absorption of beta-carotene, lycopene and lutein in men and women following a standard meal: response curves in the triacylglycerol-rich lipoprotein fraction. Br J Nutr. (1998) 79:149–59. 10.1079/bjn19980026 9536859

[31] Ross AC. Vitamin A: physiology, dietary sources, and requirements, in *Encyclopedia of Human Nutrition*. Elsevier. 333–9. 10.1016/B978-0-12-375083-9.00273-7

[32] de Pee S West CE. Dietary carotenoids and their role in combating vitamin A deficiency: a review of the literature. Eur J Clin Nutr. (1996) 50(Suppl 3) :S38–53. 8841773

[33] Borel P. Genetic variations involved in interindividual variability in carotenoid status. Mol Nutr Food Res. (2012) 56:228–40. 10.1002/mnfr.201100322 21957063

[34] van Bennekum A Werder M Thuahnai ST Han C-H Duong P Williams DL. Class B scavenger receptor-mediated intestinal absorption of dietary beta-carotene and cholesterol. Biochemistry. (2005) 44:4517–517:10.1021/bi0484320 15766282

[35] Borel P Moussa M Reboul E Lyan B Defoort C Vincent-Baudry S. Human plasma levels of vitamin E and carotenoids are associated with genetic polymorphisms in genes involved in lipid metabolism. J Nutr. (2007) 137:2653–9. 10.1093/jn/137.12.2653 18029479

[36] dela Seña C Riedl KM Narayanasamy S Curley RW Schwartz SJ Harrison EH. The human enzyme that converts dietary provitamin A carotenoids to vitamin A is a dioxygenase. J Biol Chem. (2014) 289:13661–6. 10.1074/jbc.M114.557710 24668807 PMC4036370

[37] Paik J During A Harrison EH Mendelsohn CL Lai K Blaner WS. Expression and characterization of a murine enzyme able to cleave beta-carotene. The formation of retinoids. J Biol Chem. (2001) 276:32160–8. 10.1074/jbc.M010086200 11418584

[38] Leung WC Hessel S Meung C Flint J Oberhauser V Tourniaire F. Two common single nucleotide polymorphisms in the gene encoding beta-carotene 15,15m-monoxygenase alter beta-carotene metabolism in female volunteers. FASEB J. (2009) 23:1041–53. 10.1096/fj.08-121962 19103647

[39] van Helden YGJ Heil SG van Schooten FJ Kramer E Hessel S Amengual J. Knockout of the Bcmo1 gene results in an inflammatory response in female lung, which is suppressed by dietary beta-carotene. Cell Mol Life Sci. (2010) 67:2039–56. 10.1007/s00018-010-0341-7 20372966 PMC2877315

[40] Ferrucci L Perry JRB Matteini A Perola M Tanaka T Silander K. Common variation in the beta-carotene 15,15o-monooxygenase 1 gene affects circulating levels of carotenoids: a genome-wide association study. Am J Hum Genet. (2009) 84:123–33. 10.1016/j.ajhg.2008.12.019 19185284 PMC2668002

[41] Lietz G Oxley A Leung W Hesketh J. Single nucleotide polymorphisms upstream from the β-carotene 15,15'-monoxygenase gene influence provitamin A conversion efficiency in female volunteers. J Nutr. (2012) 142:161S−5S. 10.3945/jn.111.140756 22113863

[42] Hendrickson SJ Hazra A Chen C Eliassen AH Kraft P Rosner BA. β-Carotene 15,15'-monooxygenase 1 single nucleotide polymorphisms in relation to plasma carotenoid and retinol concentrations in women of European descent. Am J Clin Nutr. (2012) 96:1379–89. 10.3945/ajcn.112.034934 23134893 PMC3497927

[43] Graßmann S Pivovarova-Ramich O Henze A Raila J Ampem Amoako Y King Nyamekye R. SNP rs6564851 in the BCO1 gene is associated with varying provitamin a plasma concentrations but not with retinol concentrations among adolescents from rural Ghana. Nutrients. (2020) 12:1786. 10.3390/nu12061786 32560166 PMC7353293

[44] Yabuta S Urata M Wai Kun RY Masaki M Shidoji Y. Common SNP rs6564851 in the BCO1 gene affects the circulating levels of β-carotene and the daily intake of carotenoids in healthy Japanese women. PLoS ONE. (2016) 11:e0168857. 10.1371/journal.pone.0168857 28005968 PMC5179075

[45] He F Xiao R-D Lin T Xiong W-M Xu Q-P Li X. Dietary patterns, BCMO1 polymorphisms, and primary lung cancer risk in a Han Chinese population: a case-control study in Southeast China. BMC Cancer. (2018) 18:445. 10.1186/s12885-018-4361-2 29673335 PMC5909209

[46] Lindqvist A Sharvill J Sharvill DE Andersson S. Loss-of-function mutation in carotenoid 15,15'-monooxygenase identified in a patient with hypercarotenemia and hypovitaminosis A. J Nutr. (2007) 137:2346–50. 10.1093/jn/137.11.2346 17951468

[47] Sharvill DE. Familial hypercarotinaemia and hypovitaminosis A. Proc R Soc Med. (1970) 63:605–6. 5453458 10.1177/003591577006300618 PMC1811589

[48] Wu L Guo X Wang W Medeiros DM Clarke SL Lucas EA. Molecular aspects of β, β-carotene-9', 10'-oxygenase 2 in carotenoid metabolism and diseases. Exp Biol Med (Maywood). (2016) 241:1879–87. 10.1177/1535370216657900 27390265 PMC5068469

[49] Reboul E Berton A Moussa M Kreuzer C Crenon I Borel P. Pancreatic lipase and pancreatic lipase-related protein 2, but not pancreatic lipase-related protein 1, hydrolyze retinyl palmitate in physiological conditions. Biochim Biophys Acta. (2006) 1761:4–10. 10.1016/j.bbalip.2005.12.013 16497549

[50] Lowe ME Rosenblum JL Strauss AW. Cloning and characterization of human pancreatic lipase cDNA. J Biol Chem. (1989) 264:20042–8 2479644

[51] Behar DM Basel-Vanagaite L Glaser F Kaplan M Tzur S Magal N. Identification of a novel mutation in the PNLIP gene in two brothers with congenital pancreatic lipase deficiency. J Lipid Res. (2014) 55:307–12. 10.1194/jlr.P041103 24262094 PMC3886669

[52] Blaner WS Brun P-J Calderon RM Golczak M. Retinol-binding protein 2 (RBP2): biology and pathobiology. Crit Rev Biochem Mol Biol. (2020) 55:197–218. 10.1080/10409238.2020.1768207 32466661 PMC7593873

[53] Crow JA Ong DE. Cell-specific immunohistochemical localization of a cellular retinol-binding protein (type two) in the small intestine of rat. Proc Natl Acad Sci USA. (1985) 82:4707–11. 10.1073/pnas.82.14.4707 3860818 PMC390973

[54] Reboul E. Absorption of vitamin A and carotenoids by the enterocyte: focus on transport proteins. Nutrients. (2013) 5:3563–81. 10.3390/nu5093563 24036530 PMC3798921

[55] Takase S Suruga K Goda T. Regulation of vitamin A metabolism-related gene expression. Br J Nutr. (2000) 84(Suppl 2):S217–21. 10.1079/096582197388572 11242473

[56] Thompson DA Li Y McHenry CL Carlson TJ Ding X Sieving PA Apfelstedt-Sylla E. Mutations in the gene encoding lecithin retinol acyltransferase are associated with early-onset severe retinal dystrophy. Nat Genet. (2001) 28:123–4. 10.1038/88828 11381255

[57] SA Humbert G Surget M-O Bazalgette C Bazalgette C Arnaud B. Screening genes of the retinoid metabolism: novel LRAT mutation in leber congenital amaurosis. Am J Ophthalmol. (2006) 142:702–4. 10.1016/j.ajo.2006.04.057 17011878

[58] Orland MD Anwar K Cromley D Chu C-H Chen L Billheimer JT. Acyl coenzyme A dependent retinol esterification by acyl coenzyme A: diacylglycerol acyltransferase 1. Biochim Biophys Acta. (2005) 1737:76–82. 10.1016/j.bbalip.2005.09.00 16214399

[59] Yen CLE Monetti M Burri BJ Farese RV. The triacylglycerol synthesis enzyme DGAT1 also catalyzes the synthesis of diacylglycerols, waxes, and retinyl esters. J Lipid Res. (2005) 46:1502–11. 10.1194/jlr.M500036-JLR200 15834126

[60] Wongsiriroj N Piantedosi R Palczewski K Goldberg IJ Johnston TP Li E Blaner WS. The molecular basis of retinoid absorption: a genetic dissection. J Biol Chem. (2008) 283:13510–9. 10.1074/jbc.M800777200 18348983 PMC2376245

[61] Arne JM Widjaja-Adhi MAK Hughes T Huynh KW Silvaroli JA Chelstowska S. Allosteric modulation of the substrate specificity of acyl-CoA wax alcohol acyltransferase 2. J Lipid Res. (2017) 58:719–30. 10.1194/jlr.M073692 28096191 PMC5392747

[62] Haas J Frese KS Park YJ Keller A Vogel B Lindroth AM. Alterations in cardiac DNA methylation in human dilated cardiomyopathy. EMBO Mol Med. (2013) 5:413–29. 10.1002/emmm.20120155 23341106 PMC3598081

[63] Harrison EH Gad MZ Ross AC. Hepatic uptake and metabolism of chylomicron retinyl esters: probable role of plasma membrane/endosomal retinyl ester hydrolases. J Lipid Res. (1995) 36:1498–506. 7595074

[64] Blomhoff R Helgerud P Rasmussen M Berg T Norum KR. *In vivo* uptake of chylomicron [3H]retinyl ester by rat liver: evidence for retinol transfer from parenchymal to non-parenchymal cells. Proc Natl Acad Sci USA. (1982) 79:7326–30. 10.1073/pnas.79.23.7326 6961410 PMC347332

[65] Goodman DW Huang HS Shiratori T. Tissue distribution and metabolism of newly absorbed vitamin a in the rat. J Lipid Res. (1965) 6:390–6. 14336210

[66] Hussain MM Mahley RW Boyles JK Fainaru M Brecht WJ Lindquist PA. Chylomicron-chylomicron remnant clearance by liver and bone marrow in rabbits. Factors that modify tissue-specific uptake. J Biol Chem. (1989) 264:9571–82. 2722852

[67] Merrill AH Bowman BB Preusch PC. Mechanistic aspects of vitamin and coenzyme utilization and function: a symposium in recognition of the distinguished career of Donald B. McCormick. J Nutr. (2000) 130:321S−2S. 10.1093/jn/130.2.321S 10721896

[68] Quadro L Hamberger L Colantuoni V Gottesman ME Blaner WS. Understanding the physiological role of retinol-binding protein in vitamin A metabolism using transgenic and knockout mouse models. Mol Aspects Med. (2003) 24:421–30. 10.1016/s0098-2997(03)00038-4 14585313

[69] Kawaguchi R Yu J Honda J Hu J Whitelegge J Ping P. membrane receptor for retinol binding protein mediates cellular uptake of vitamin A. Science. (2007) 315:820–5. 10.1126/science.1136244 17255476

[70] Kennedy OJ Pirastu N Poole R Fallowfield JA Hayes PC Grzeszkowiak EJ. Coffee consumption and kidney function: a mendelian randomization study. Am J Kidney Dis. (2020) 75:753y Dis 10.1053/j.ajkd.2019.08.025 31837886

[71] Wuttke M Li Y Li M Sieber KB Feitosa MF Gorski M. A catalog of genetic loci associated with kidney function from analyses of a million individuals. Nat Genet. (2019) 51:957tog of genet1038/s41588-019-0407-x 31152163 10.1038/s41588-019-0407-x PMC6698888

[72] Weafer J Gray JC Hernandez K Palmer AA MacKillop J de Wit H. Hierarchical investigation of genetic influences on response inhibition in healthy young adults. Exp Clin Psychopharmacol. (2017) 25:512armac 10.1037/pha0000156 29251981 PMC5737791

[73] Han X Qassim A An J Marshall H Zhou T Ong J-S. Genome-wide association analysis of 95 of genetic influences on response inhibition in healthy young adults. adults. inhHum Mol Genet. (2019) 28:3680–680:10.1093/hmg/ddz193 31809533

[74] Kichaev G Bhatia G Loh P-R Gazal S Burch K Freund MK. Leveraging polygenic functional enrichment to improve GWAS power. Am J Hum Genet. (2019) 104:65 Gene 10.1016/j.ajhg.2018.11.008 30595370 PMC6323418

[75] Casey J Kawaguchi R Morrissey M Sun H McGettigan P Nielsen JE. First implication of STRA6 mutations in isolated anophthalmia, microphthalmia, and coloboma: a new dimension to the STRA6 phenotype. Hum Mutat. (2011) 32:1417–417:10.1002/humu.21590 21901792 PMC3918001

[76] Richardson R Sowden J Gerth-Kahlert C Moore AT Moosajee M. Clinical utility gene card for: non-syndromic microphthalmia including next-generation sequencing-based approaches. Eur J Hum Genet. (2017) 25:201. 10.1038/ejhg.2016.201 28098148 PMC5386421

[77] Golzio C Martinovic-Bouriel J Thomas S Mougou-Zrelli S Grattagliano-Bessieres B Bonniere M. Matthew-Wood syndrome is caused by truncating mutations in the retinol-binding protein receptor gene STRA6. Am J Hum Genet. (2007) 80:1179–179:10.1086/518177 17503335 PMC1867105

[78] White T Lu T Metlapally R Katowitz J Kherani F Wang T-Y. Identification of STRA6 and SKI sequence variants in patients with anophthalmia/microphthalmia. *Mol Vis*. (2008) 14:2458–458:Srinivasan BS, Doostzadeh J, Absalan F, Mohandessi S, Jalili R, Bigdeli S, et al. Whole genome survey of coding SNPs reveals a reproducible pathway determinant of Parkinson disease. Hum Mutat. (2009) 30:228tenom 10.1002/humu.20840 18853455 PMC2793088

[79] Nair AK Sugunan D Kumar H Anilkumar G. Case-control analysis of SNPs in GLUT4, RBP4 and STRA6: association of SNPs in STRA6 with type 2 diabetes in a South Indian population. PLoS ONE. (2010) 5:e11444. 10.1371/journal.pone.0011444 20625434 PMC2897881

[80] Richards S Aziz N Bale S Bick D Das S Gastier-Foster J. Standards and guidelines for the interpretation of sequence variants: a joint consensus recommendation of the American college of medical genetics and genomics and the association for molecular pathology. Genet Med. (2015) 17:405df me 10.1038/gim.2015.30 25741868 PMC4544753

[81] Huang H-W Liang B-Y Li Y-X. Association of Polymorphisms in STRA6 and RARRES2 genes with type 2 diabetes in Southern Han Chinese. Biomed Res Int. (2016) 2016:6589793. 10.1155/2016/6589793 27446956 PMC4947507

[82] Hu S Yan J You Y Yang G Zhou H Li X. Association of polymorphisms in STRA6 gene with gestational diabetes mellitus in a Chinese Han population. Medicine (Baltimore). (2019) 98:e14885. 10.1097/MD.0000000000014885 30882700 PMC6426506

[83] Mu-Herneal dFernn JB DJB -Ch DMondrag-Fonseca O Mayns-Lobo Y Ortega A. STRA6 polymorphisms are associated with EGFR mutations in locally-advanced and metastatic non-small cell lung cancer patients. Front Oncol. (2020) 10:579561. 10.3389/fonc.2020.579561 33324556 PMC7723324

[84] Blaner WS O WS Olung Wongsiriroj N Kluwe J DJ D J D DM Jiang H. Hepatic stellate cell lipid droplets: a specialized lipid droplet for retinoid storage. Biochim Biophys Acta. (2009) 1791:46709s 10.1016/j.bbalip.2008.11.001 19071229 PMC2719539

[85] Randolph RK Ross AC. Vitamin A status regulates hepatic lecithin: retinol acyltransferase activity in rats. J Biol Chem. (1991) 266:16453–645Dawson HD, Yamamoto Y, Zolfaghari R, Rosales FJ, Dietz J, Shimada T, et al. Regulation of hepatic vitamin A storage in a rat model of controlled vitamin A status during aging. J Nutr. (2000) 130:1280–28 10.1093/jn/130.5.1280 1885578

[86] Zolfaghari R Ross AC. Lecithin:retinol acyltransferase from mouse and rat liver. CDNA cloning and liver-specific regulation by dietary vitamin a and retinoic acid. J Lipid Res. (2000) 41:2024–024:10.1016/S0022-2275(20)32364-6 11108736

[87] Taschler U Schreiber R Chitraju C Grabner GF Romauch M Wolinski H. Adipose triglyceride lipase is involved in the mobilization of triglyceride and retinoid stores of hepatic stellate cells. Biochim Biophys Acta. (2015) 1851:93715s A 10.1016/j.bbalip.2015.02.017 25732851 PMC4408194

[88] Pirazzi C Valenti L Motta BM Pingitore P Hedfalk K Mancina RM. PNPLA3 has retinyl-palmitate lipase activity in human hepatic stellate cells. Hum Mol Genet. (2014) 23:4077–077:10.1093/hmg/ddu121 24670599 PMC4082369

[89] Fischer J Lefcher C Morava E Mussini J-M Lafor L P Negre-Salvayre A. The gene encoding adipose triglyceride lipase (PNPLA2) is mutated in neutral lipid storage disease with myopathy. Nat Genet. (2007) 39:28etted 10.1038/ng1951 17187067

[90] Akiyama M Sakai K Ogawa M McMillan JR Sawamura D Shimizu H. Novel duplication mutation in the patatin domain of adipose triglyceride lipase (PNPLA2) in neutral lipid storage disease with severe myopathy. Muscle Nerve. (2007) 36:856erve 10.1002/mus.20869 17657808

[91] Lin P Li W Wen B Zhao Y Fenster DS Wang Y. Novel PNPLA2 gene mutations in Chinese Han patients causing neutral lipid storage disease with myopathy. J Hum Genet. (2012) 57:679netio 10.1038/jhg.2012.84 22832386

[92] Reilich P Horvath R Krause S Schramm N Turnbull DM Trenell M. The phenotypic spectrum of neutral lipid storage myopathy due to mutations in the PNPLA2 gene. J Neurol. (2011) 258:1987–9878. 10.1007/s00415-011-6055-4 21544567

[93] Kovarova M KM Kova MI KI Kova M A Machicao F HF Hc H-U Schleicher E. The genetic variant I148M in PNPLA3 is associated with increased hepatic retinyl-palmitate storage in humans. J Clin Endocrinol Metab. (2015) 100:E156800:in 10.1210/jc.2015-2978 26439088

[94] Mondul A Mancina RM Merlo A Dongiovanni P Rametta R Montalcini T. PNPLA3 I148M Variant influences circulating retinol in adults with non-alcoholic fatty liver disease or obesity. J Nutr. (2015) 145:1687–6875. 10.3945/jn.115.210633 26136587 PMC4516767

[95] GTEx Consortium. Human genomics. The Genotype-Tissue Expression (GTEx) pilot analysis: multitissue gene regulation in humans. Science. (2015) 348:648–60. 10.1126/science.1262110 25954001 PMC4547484

[96] GTEx Consortium. The genotype-tissue expression (GTEx) project. Nat Genet. (2013) 45:580–5. 10.1038/ng.2653 23715323 PMC4010069

[97] Zhu Z Zhang F Hu H Bakshi A Robinson MR Powell JE. Integration of summary data from GWAS and eQTL studies predicts complex trait gene targets. Nat Genet. (2016) 48:481ttio 10.1038/ng.3538 27019110

[98] Jansen RC Nap JP. Genetical genomics: the added value from segregation. Trends Genet. (2001) 17:388eneth 10.1016/s0168-9525(01)02310-1 11418218

[99] Galicia L Grajeda R de Romade DL. Nutrition situation in Latin America and the Caribbean: current scenario, past trends, and data gaps. Rev Panam Salud Publica. (2016) 40:104blicaWirth JP, Petry N, Tanumihardjo SA, Rogers LM, McLean E, Greig A, Garrett GS, Klemm RDW, Rohner F. Vitamin A supplementation programs and country-level evidence of vitamin A deficiency. Nutrients (2017) 9:190. 10.3390/nu9030190 28245571 PMC5372853

[100] West KP. Vitamin A deficiency disorders in children and women. Food Nutr Bull. (2003) 24:S78724:3ull Bul1177/15648265030244S204 17016949 10.1177/15648265030244S204

[101] World Health Organization. WHO Global Prevalence of Vitamin A Deficiency in Populations at Risk 1995–2005. WHO Global Database on Vitamin A Deficiency. Washington, DC: World Health Organization (2009).

[102] Tanumihardjo SA Russell RM Stephensen CB Gannon BM Craft NE Haskell MJ Lietz G Schulze K Raiten DJ. Biomarkers of Nutrition for Development (BOND)-Vitamin A Review. J Nutr. (2016) 146:1816S−48S. 10.3945/jn.115.229708 27511929 PMC4997277

